# Health-related quality of life among patients with end-stage renal disease undergoing hemodialysis in Ethiopia: a cross-sectional survey

**DOI:** 10.1186/s12955-023-02117-x

**Published:** 2023-04-17

**Authors:** Mignote Hailu Gebrie, Hussen Mekonnen Asfaw, Workagegnehu Hailu Bilchut, Helena Lindgren, Lena Wettergren

**Affiliations:** 1 College of Medicine and Health Sciences, School of Nursing, University of Gondar, Gondar, Ethiopia; 2 College of Health Sciences, School of Nursing & Midwifery, Department of Nursing, Addis Ababa University, Addis Ababa, Ethiopia; 3 College of Medicine and Health Sciences, School of Medicine, Department of Internal Medicine, University of Gondar, Gondar, Ethiopia; 4 Department of Women’s and Children’s Health, Division of Reproductive Health, Karolinska Institutet, Solna, Sweden; 5 Sophiahemmet University, Stockholm, Sweden; 6 Department of Public Health and Caring Sciences, Uppsala University, Solna, Sweden

**Keywords:** End-stage renal disease, Hemodialysis, Health-related quality of life, KDQOL-36, Kidney failure, Addis Ababa, Ethiopia

## Abstract

**Background:**

Measurement of health-related quality of life (HRQOL) enables identification of treatment-related side effects of a disease. Such aspects may negatively impact on patients’ lives and should be taken into consideration in medical decision-making. In sub-Saharan Africa, research from the perspective of patients with chronic kidney disease is scarce, and it is almost non-existent in patients undergoing hemodialysis. We aimed to determine HRQOL among end-stage renal disease patients undergoing maintenance hemodialysis in Ethiopia and to identify factors associated with HRQOL.

**Methods:**

A multi-center cross-sectional study was conducted in Addis Ababa, Ethiopia directed to all patients receiving hemodialysis due to kidney failure at 11 randomly-selected government and private hospitals/dialysis centers in the capital of Ethiopia. Data were collected by trained nurses using the KDQOL-36 instrument with five subscales measuring generic and disease-specific HRQOL. Study-specific items were used to collect socio-demographic and clinical data. Factors associated with HRQOL were examined using multivariable linear regression models.

**Results:**

Four hundred eighty-one patients completed the survey through face-to-face interviews (response rate 96%; mean age 45.34 ± 14.67). The mean scores of the subscales ranged from 25.6 to 66.68 (range 0–100), with higher scores reflecting better health. Factors associated with low HRQOL included older age, female sex, no formal education, poor medication adherence, > 2 hemodialysis sessions/week, lower body mass index (< 18.5), longer duration of hemodialysis treatment (≥ 12 months), and poor social support.

**Conclusion:**

Patients with kidney failure undergoing hemodialysis in Addis Ababa, Ethiopia, had low HRQOL across all subscales compared to previous studies. Therefore, the implementation of guidelines is crucial to improve patients’ adherence to their prescribed medications. Furthermore, establishing patient support groups and encouraging patients to use the available support resources from family members, neighbors, and friends have the potential to improve patients’ HRQOL.

**Supplementary Information:**

The online version contains supplementary material available at 10.1186/s12955-023-02117-x.

## Background

Chronic kidney disease (CKD) is a public health problem worldwide, and the number of people who require renal replacement therapy is increasing [1, 2]. CKD is defined as either kidney damage or a glomerular filtration rate below 60 ml/min/1.73 m2 that lasts for ≥ 3 months [3, 4]. End-stage renal disease (ESRD), lately suggested to be labelled as “kidney failure” [5], is the final stage of CKD, and a serious health condition which has progressive and debilitating effects on a patient’s life [2, 6]. According to the Kidney Disease: Improving Global Outcomes (KDIGO) Working Group on the Evaluation and Management of Chronic Kidney Disease’s revised 2012 classification [7], CKD is classified based on cause, glomerular filtration rate (G1, G2, G3a, G3b, G4, and G5), and albuminuria category (A1, A2, and A3).

The leading cause of CKD is diabetes mellitus, followed by hypertension, glomerulonephritis, polycystic kidney disease, tubule–interstitial diseases, and genetic disorders [8, 9]. A systematic analysis conducted for the Global Burden of Disease Study reported that the burden of CKD in 2017 was much higher in sub-Saharan Africa than would be expected for its level of development [10].

The incidence and prevalence of ESRD continue to increase worldwide [2, 6]. However, the burden in Africa is not established due to inadequate registration systems, with the highest registered prevalence in Tunisia and Egypt, at 713 and 669 per million population, respectively [11]. Although the true burden of ESRD in Sub-Saharan Africa is unknown, it is assumed to be high, with risk factors including both communicable and non-communicable diseases [12].

Renal replacement therapy—including hemodialysis, peritoneal dialysis and renal transplantation—is the primary treatment for patients with ESRD [13]. Renal transplantation is the best form of therapy for most patients [2]. Hemodialysis is the most commonly utilized treatment method in low-resource countries, although its provision is challenging [2, 14]. Moreover, while hemodialysis has improved patient survival, patients undergoing hemodialysis suffer from a range of health problems, including sleep disorders, peripheral neuropathy, fatigue, stress, emotional distress, cognitive impairment, pain, and sexual dysfunction [15, 16]. It is also time-intensive, expensive, and often results in loss of freedom, dependence on caregivers, disruption of family and social life, and reduced or complete loss of financial income [17].

According to the Global Burden of Disease Study 2017, [10], Ethiopia had close to 5 million estimated cases of CKD and approximately 9000 deaths. The age-standardized rate of CKD was reported to be 9376 per 100,000 population. The incidence of CKD is increasing in Ethiopia, due to the rising prevalence of hypertension and diabetes mellitus, which are well-known risk factors for CKD [18]. Currently, hemodialysis is the only type of dialysis offered to ESRD patients in Ethiopia. The vast majority of ESRD patients receive palliative care because they cannot afford or access a dialysis facility. Excluding the cost of medications for comorbidities, and other ancillary costs, the average cost of a single hemodialysis session in the private sector is 2300 Ethiopian birr (equivalent to USD 51) [19]. Dialysis for the treatment of ESRD has been available for less than two decades in Ethiopia. It is mainly accessible to rich people who are living in Addis Ababa and other large cities who can afford the high cost of dialysis and other ESRD-related care [20].

In patients with ESRD for whom curative treatment is not a realistic goal, the primary objective of care is often enhancing health-related quality of life, which is emphasized in the current care provision [14, 21]. Health-related quality of life (HRQOL) is a broad multidimensional concept including at least three domains: physical, psychological, and social functioning [22]. HRQOL is subjective and focuses on the impact of a perceived health state on a patient’s ability to live a fulfilling life. Its measurement has significance for the evaluation and follow-up of chronic patients involved in lifelong intervention services like hemodialysis [14, 23]. In order to provide holistic care, and in keeping with World Health Organization’s (WHO) definition of health as not only the absence of disease and infirmity but also the presence of physical, mental, and social well-being, HRQOL has become increasingly important [24].

Globally, several studies have reported that ESRD patients undergoing hemodialysis have poor HRQOL [25–30]. Several factors, clinical, socio-demographic and behavioural, have shown to be associated to HRQOL including age, sex, educational status, employment status and comorbidities. However, few studies have investigated HRQOL in patients with ESRD undergoing hemodialysis in Africa [31–33] and only three in Ethiopia [34–36]. Two of them included all patients with CKD (stage 1 to 5) [35, 36]. Therefore, the aim of the present study was to determine HRQOL, and factors associated with HRQOL, among patients with ESRD undergoing maintenance hemodialysis in Ethiopia.

## Methods

### Study design and setting

A multi-center institution-based cross-sectional study was employed during a three-month period from February to April 2021. The study was conducted in both governmental and private hospitals and dialysis centers located in Addis Ababa. According to data obtained from Addis Ababa’s city health bureau, there were 4 governmental and 16 private hospitals providing hemodialysis service in the city. Data were collected from 11 randomly selected (2 governmental and 9 private) hospitals and dialysis centers.

### Sample

A probability sampling strategy was applied by randomly selected 11 (2 governmental and 9 private) hospitals/dialysis centers in the larger area of the capital in Ethiopia. The study covered all eligible patients with ESRD undergoing hemodialysis in the 11 selected hospitals/dialysis centers. Inclusion criteria were patients ≥ 18 years with ESRD (according to medical records) who had been on regular maintenance hemodialysis for at least three months. Patients with a documented diagnoses of Alzheimer’s disease, dementia or other cognitive impairments were excluded from the study (n = 3).

### Measures

#### The KDQOL-36 instrument

The KDQOL-36 is an established self-report instrument that was developed for individuals with kidney disease and those on dialysis [37]. KDQOL-36 includes the Medical Outcomes Study’s 12-Item Short-Form Health Survey (SF-12), one of the most widely used generic measures of HRQOL, and 24 disease-specific items related to kidney disease. The SF-12 includes two domains: the Physical Component Summary (PCS) and the Mental Component Summary (MCS). The disease-specific items encompass three domains, which assess Burden of Kidney Disease (BKD), Symptoms and Problems of Kidney Disease (SPKD), and Effects of Kidney Disease (EKD). The BKD scale has four items (e.g., “I feel like a burden to my family”), with five response options that range from “definitely true” to “definitely false”. The SPKD scale has 12 items, each representing a symptom or side effect of kidney disease (e.g., “muscle soreness”), with five response options ranging from “not at all bothered” to “extremely bothered”. The EKD scale has eight items (e.g., “fluid restriction”), with the same response options as the Symptoms/Problems subscale. Each scale’s raw scores are converted to a possible range of 0–100, with higher scores reflecting better health and fewer symptoms. Mean scores for the five main domains were generated using the Hays algorithm [38]. The original English version of the KDQOL-36 has been shown to be reliable and valid for assessing hemodialysis patients’ HRQOL in various nations [29, 37]. The original KDQOL-36 instrument was translated into the local language (Amharic), and the preliminary Amharic version was psychometrically evaluated and found to be reliable and valid for an Ethiopian context [39]. Cronbach alpha in the present study ranged from 0.78 (Burden of kidney disease) to 0.91 (Symptoms of kidney disease).

#### The simplified medication adherence questionnaire

Adherence to medication was assessed using the six-item Simplified Medication Adherence Questionnaire [40]. A total score of 12 is considered to reflect adherence to medication, while scores < 12 indicate non-adherence. The tool has demonstrated acceptable validity and reliability in an Amharic-speaking population [40]. Cronbach alpha in the present study was 0.82.

#### The oslo social support scale

The Oslo Social Support Scale was used to measure the participants’ perceived social support [41]. The scale consists of three items assessing the patient’s number of close contacts, their sense of concern from other people, and their relationship with their neighbors, with a focus on the accessibility of practical help. The responses are scored from 3 to 14, with higher values reflecting a stronger level of social support. The level of social support is classified into three groups based on the total score (poor = 3–8, moderate = 9–11, and strong = 12–14). The Oslo Social Support Scale has been used in a number of studies and found to be practicable, with strong predictive and convergent validity [42]. Cronbach alpha in the present study was 0.71.

Study-specific items were used to collect socio-demographic data (age, sex, education, residence, marital status, family size, occupation, and monthly family income). Clinical characteristics were extracted from patients’ medical records. Behavioral factors were assessed using 17 study-specific items (supplementary material).

### Procedure

Data were collected after obtaining permission from each hospital’s administrative body. Patients were identified through patient lists generated by the head nurse of each dialysis unit. Potential participants were approached during treatment and given written and oral information about the study’s aims and procedures. Participants were informed that non-participation would not affect care or treatment in any way. Data were collected by face-to-face interviews by nurses with baccalaureate degrees working outside the individual hospital where data were collected. Data collectors and supervisors were trained on study procedures, questionnaires, data collection techniques, and study ethics for three days. Clinical data were extracted from individual patients’ medical records.

### Data processing and analysis

STATA version 14 software was used for statistical analysis [43], and the KDQOLTm-36 Excel scoring program (version 2.0) was used to compute the scale scores (http://www.rand.org/health/surveys_tools/kdqol.html). Mean scores and standard deviations of the five subscales were calculated [38]. Multivariable linear regression was conducted to identify associations between socio-demographic factors (age, sex, marital status, education, occupation, monthly family income, family size, funding/sponsor, and distance from hospital/dialysis center), clinical factors (comorbidities, number of hemodialysis sessions per week, vascular access type, duration of hemodialysis, family history of CKD, medication adherence, anemia, and body mass index [BMI]), behavioral factors (khat chewing, cigarette smoking, and alcohol use) and social support for each HRQOL domain. We performed a model fitness test to look for any clustering effects at the hospital level, but there were none in any of the KDQOL-36 instrument’s domains. We checked the regression model’s assumptions by inspecting the plots of residuals versus predicted values to check the normality, linearity, and homoscedasticity of our data. Multicollinearity was checked by calculating the mean VIF (variance inflation factor). All variables were entered one at a time, and a stepwise backward model building technique was used where variables with the largest p-value were removed step-by-step to construct the final model. Results are reported as the estimated regression coefficient ‘β’. P-values < 0.05 were considered statistically significant.

## Results

### Socio-demographic characteristics

A total of 502 patients with ESRD undergoing hemodialysis were approached, and 481 patients completed the survey (for a response rate of 96%). The age range of the participants was between 18 and 70 years (mean 45.34 ± 14.67). About one fourth of the patients lacked formal education (16.2%) or had completed primary school (10.4%), while a majority had undergone secondary school (27.7%) or at least reached a certificate level (45.7%). Almost half of the participants were unemployed 239 (49.7%). Only 93 (19.3%) of the study participants had funding for their dialysis treatment (Table 1).

**Table 1:** Socio-demographic characteristics of patients with ESRD undergoing hemodialysis in Addis Ababa, Ethiopia (n = 481)

Variables	Category	Numbers (n)	Percent (%)
Sex	Men	307	63.8
	Women	174	36.2
Age (years), Mean (SD)	45.34 (SD ± 14.67)		
Residence	In Addis Ababa	391	81.3
	Outside Addis Ababa	90	18.7
Marital status	Ever married	358	74.4
	Single	123	25.6
Educational status	No formal education	78	16.2
	Primary	50	10.4
	Secondary	133	27.7
	Certificate and above	220	45.7
Monthly family income	≤ 6500 birr (123 USD)	252	52.4
	> 6500 birr (123 USD)	229	47.6
Occupation	Employed	242	50.3
	Unemployed	239	49.7
Funding/Sponsor	Yes	93	19.3
	No	388	80.7
Family Size	Single household	8	1.7
	2–4	177	36.8
	≥ 5	296	61.5
Distance to hospital/center, km	≤ 10	280	58.2
	> 10	201	41.8
Hospital type	Governmental	63	13.1
	Private	418	86.9

### Clinical characteristics

In this study, hypertension 167 (34.7%) and diabetes mellitus 160 (33.3%) were the most common underlying diseases of ESRD patients on hemodialysis. The duration of hemodialysis ranged from 4 to 96 months. Approximately half of the respondents were adherent to their medication, and one-third of respondents’ ratings indicated that they had strong social support (Table 2).

**Table 2:** Clinical characteristics of patients with ESRD undergoing hemodialysis in Addis Ababa, Ethiopia (n = 481)

Variables	Category	Numbers (n)	Percent (%)
Comorbidities	Yes	445	92.5
	No	36	7.5
Number of hemodialysis sessions per week	1–2	295	61.3
	3	186	38.7
Vascular access type	AV fistula	395	82.1
	AV graft	35	7.3
	Permanent catheter	43	8.9
	Temporary catheter	8	1.7
Duration of hemodialysis	< 12 months	216	44.9
	12–36 months	172	35.8
	≥ 37 months	93	19.3
Family history of CKD	Yes	25	5.2
	No	456	94.8
Medication adherence ^a^	Adhered	255	53.0
	Not adhered	226	47.0
Anemia (serum hemoglobin < 12 g/dl)	Yes	409	85.0
	No	72	14.9
BMI	Underweight	104	21.6
	Normal weight	307	63.8
	Overweight	57	11.9
	Obese	13	2.7

CKD: chronic kidney disease, AV: Arterio-venous, BMI: body mass index

^a^Simplified Medication Adherence Questionnaire ≥ 12 = adherent, < 12 = non-adherent to medication

### Behavioral factors

Regarding behavioral factors, 57 (11.9%), 74 (15.4%), and 145 (30.1%) of participants had ever smoked cigarettes, ever chewed khat, or ever consumed alcohol, respectively (Table 3).

**Table 3:** Behavioral factors among patients with ESRD undergoing hemodialysis in Addis Ababa, Ethiopia (n = 481)

Variables	Category	Numbers (n)	Percent (%)
Ever smoked cigarettes	Yes	54	11.9
	No	427	88.1
Current smoker	Yes	2	3.6
	No	52	96.4
Family member smokes cigarette	Yes	44	9.1
	No	437	90.9
Ever chewed khat	Yes	74	15.4
	No	407	84.6
Current chewer	Yes	3	0.6
	No	71	14.8
Ever consumed alcohol	Yes	145	30.1
	No	336	69.9
Alcohol consumption less than once a month	Yes	3	0.6
	No	8	1.7

### Health-related quality of life

The mean scores of the five domains of the KDQOL-36 instrument ranged from 25.6 for the BKD scale to 66.68 for the SPKD scale (Table 4). Itchy skin (pruritus) was one of the most prevalent symptoms in our study (SPKD subscale), with 84% of patients reporting at least a moderate level followed by the symptoms “fainting or dizziness” (83.7%) and feeling “washed out or drained” (81%).

**Table 4:** Mean scores of the domains of the KDQOL-36 rated by patients undergoing hemodialysis in Addis Ababa, Ethiopia (n = 481)

Subscales	Mean (SD)	95% CI
Symptom/problem list	66.68 (20.31)	64.72–68.43
Effects of kidney disease	51.93 (20.53)	50.11–53.80
Burden of kidney disease	25.60 (23.16)	23.55–27.81
SF-12 Physical Component Summary	35.45 (8.08)	34.76–36.17
SF-12 Mental Component Summary	37.48 (11.34)	36.49–38.54

CI, Confidence interval

**Table 5:** Socio-demographic characteristics associated with health-related quality of life domains among haemodialysis patients in Addis Ababa, Ethiopia (n = 481)

Factors	PCS β^a^ (95% CI)	MCS β^a^ (95%CI)	Symptoms β^a^ (95%CI)	Effects β^a^ (95%CI)	Burden β^a^ (95%CI)
Age	-0.15 (-0.21– -0.09)*	-0.001 (-0.1–0.08)	-0.03 (-0.19–0.13)	-0.21 (-0.37– -0.05)*	0.10 (-0.08–0.28)
Sex					
Male	Ref.	Ref.	Ref.	Ref.	Ref.
Female	-0.29 (-1.89-1.29)	-3.9(-6.26- -1.55)*	-4.08(-8.26–0.09)	-3.58(-7.73–0.57)	-3.67(-9.50–2.20)
Educational status					
No formal education	Ref.	Ref.	Ref.	Ref.	Ref.
Primary school	0.58 (-2.12–3.29)	2.91 (-1.1–6.92)	1.48 (-5.6–8.57)	3.98 (-3.08–11.03)	-0.51(-8.69–7.68)
Secondary school	1.30 (-0.93–3.54)	2.87 (-0.39–6.14)	3.46 (-2.38–9.3)	7.01(1.19–12.84)*	6.07 (0.57–12.72)
Certificate and above	3.0 (0.830–5.17)*	2.64 (-0.49–5.76)	4.59 (-1.07–10.25)	5.58 (-0.07–11.22)	8.05 (1.83–14.28)*
Marital status					
Single	Ref.	Ref.	Ref.	Ref.	Ref.
Ever married	0.05 (-1.89–2.00)	-0.36 (-3.23–2.52)	3.32 (-8.6–1.95)	-2.26 (-7.34–2.83)	-3.85 (-9.70–2.01)
Family monthly income					
≤ 6500	Ref.	Ref.	Ref.	Ref.	Ref.
> 6500	-0.49 (1.93–0.94)	0.26 (-1.85–2.36)	1.78 (-1.97–5.54)	-1.13 (-4.87–2.59)	-2.53 (-6.84–1.77)

*p-value < 0.05; Ref.: reference category; PCS: physical component summary; MCS: mental component summary score

### Factors associated with health-related quality of life

This study found that higher age, female sex, a lack of formal education, poor medication adherence, hemodialysis sessions three times a week, lower BMI, longer duration of hemodialysis treatment (≥ 12 months), and poor social support were associated with poor HRQOL (Table 5) (Additional file 1). Accordingly, the coefficients of age in the physical component summary and effects of the kidney disease subscales were β = − 0.15 and − 0.17, respectively; indicating that a unit increase in age decreases the mean scores of the physical component summary and effects of kidney disease subscales by 0.15 and 0.17, respectively. Patients who had certificate-level and above education had higher PCS (better physical health) scores and rated their kidney disease burden as lower.

Clinical characteristics associated with lower HRQOL in the EKD and SPKD subscales included longer duration of hemodialysis treatment (≥ 12 months, see Table 6) (Additional file 2). Moderate social support was strongly associated with better HRQOL in physical health domains. Adherence to subscribed medication was significantly associated with a higher HRQOL in the EKD domain. A significant negative association was found between the number of hemodialysis sessions (three times a week) and the physical health domain. A higher BMI (≥ 18.5) was strongly and consistently associated with better HRQOL across all domains. However, no statistically significant associations were found between HRQOL domains and behavioral factors, or the remaining socio-demographic and clinical variables.

**Table 6:** Associations between clinical characteristics and social support, and health-related quality of life domains among patients undergoing haemodialysis in Addis Ababa, Ethiopia (n = 481)

Factors	PCS β^a^ (95% CI)	MCS β^a^ (95%CI)	Symptoms β^a^ (95%CI)	Effects β^a^ (95%CI)	Burden β^a^ (95%CI)
Comorbidities					
Yes	Ref.	Ref.	Ref.	Ref.	Ref.
No	0.84 (-1.83–3.51)	-2.87 (-6.8–1.06)	-0.22(-7.24–6.8)	3.35(-3.6–10.33)	-6.21(-14.2–1.82)
Haemodialysis sessions per week					
1–2	Ref.	Ref.	Ref.	Ref.	Ref.
3	-2.17(-3.64– -0.70)*	0.97(-1.18–3.12)	2.15(-1.65–5.95)	-2.80(-6.59–0.98)	-2.79(-7.18–1.60)
Duration of haemodialysis treatment					
<12 months	Ref.	Ref.	Ref.	Ref.	Ref.
12–36 months	0.47(-1.17–2.10)	0.13(-2.29–2.55)	-5.79(-10.08– -1.49)*	-5.65(-9.91– -1.38)*	-9.08(-14.03– -4.13)
≥ 37 months	-1.19(-3.144–0.76)	-2.53(-5.42–0.37)	-10.48(-15.6– -5.36)*	-11.9(-16.9– -6.8)*	-9.06(-14.97– -3.15)
Medication adherence					
Adhered	Ref.	Ref.	Ref.	Ref.	Ref.
Not adhered	-0.99(-2.36–0.38)	-0.91(-2.94–1.11)	-3.56(-7.14-0.03)	-4.6(-8.17– -1.03)*	-1.78(-5.92–2.36)
BMI					
Under weight	Ref.	Ref.	Ref.	Ref.	Ref.
Normal weight	3.34(1.59–5.08)*	2.15(-0.43–4.73)	5.82(1.26–10.38) *	8.04(3.5–12.59)*	3.06(-2.22–8.34)
Overweight	3.51(0.96–6.07)*	3.45(-0.31–7.2)	4.64(-2.11–11.39)	7.78(1.11–14.44)*	2.43(-5.25–10.11)
Obese	0.07(4.39–4.53)	9.87(3.28–16.47)*	17.47(5.71–29.24)*	15.48(3.84–27.11)*	20.15(6.67–33.62)*
Social support					
Poor	Ref.	Ref.	Ref.	Ref.	Ref.
Moderate	1.84(0.24–3.43)*	-0.67(-3.03–1.69)	0.62(-3.56–4.81)	-0.58(-4.75–3.58)	0.15(-4.67–4.97)
Strong	1.68(-0.20–3.57)	-3.67(-6.46–1.87)	-3.76(-8.74–1.22)	2.67(-2.26–7.59)	4.09(-1.62–9.8)

*p-value < 0.05; Ref.: reference category; PCS: physical component summary; MCS: mental component summary score; BMI: body mass index; ^a^ Multivariable linear regression

## Discussion

A multi-center institution-based cross-sectional study was carried out among patients with ESRD undergoing hemodialysis in both governmental and private hospitals/dialysis centers in Addis Ababa, Ethiopia. The study demonstrated that patients undergoing hemodialysis rated their HRQOL as poor. Several socio-demographic and clinical characteristics of hemodialysis patients were found to be associated with both generic and kidney disease-related components of HRQOL.

The average age of the patients in our study (45 years) was broadly comparable to that of patients undergoing hemodialysis in other African countries [17, 32] and close to the mean age of patients in south-Asian countries [44, 45]. This could be explained by the fact that, in Sub-Saharan Africa and Asia, ESRD affects younger populations due to the influence of established risk factors, such as diabetes, hypertension and kidney infection [46, 47]. In support of this explanation, our data showed that 67% and 76% of diabetes and hypertensive patients, respectively, were younger than 60 years of age.

Our results showed that patients undergoing hemodialysis in Ethiopia struggle with poor physical and mental health (subscales PCS and MCS, respectively). This is in line with previous reports [30–32, 34, 48–50]; however, our mean scores (PCS and the MCS) were slightly lower than most other studies when taking the confidence intervals into account. A study from Malawi [32] with a small sample (n = 22) found higher scores of both PCS and MCS, and a single center study carried out in Colombia [30] reported higher ratings of PCS. Additionally, a large study from the US [25] reported better physical and mental health in a large sample of patients with all types of dialysis (in center hemodialysis, peritoneal dialysis, home hemodialysis, and nocturnal dialysis). Differences in sampling strategies, sample size, and response rates limit the possibility to conclude that our results are worse than previously reported. Our results underscore that this group of patients not only have serious limitations regarding physical health, but also mental health issues that merit concern.

Regarding the kidney disease-targeted part of the instrument, the BKD subscale was rated low, indicating that patients on maintenance hemodialysis experienced a huge disease burden. This finding is in line with two previous studies conducted in Ethiopia and Malawi but higher compared to a study performed in Kenya [49]. Our results were lower reflecting more frustration and interference, than those from the US, Europe (France, Germany, Italy, Spain, and the United Kingdom), and Japan [25, 48] or those studies performed in Colombia and Egypt [30, 31]. A possible explanation of this discrepancy might be related to differences in healthcare systems. The healthcare system in Ethiopia requires lengthy procedures and numerous diagnostic tests, which may frustrate the patient. In addition, restrictions on mobility, daily activities, and the risk of losing one’s job due to dialysis therapy can result in a feeling of frustration and cause dialysis patients to perceive themselves to be a burden on their families. “Itchy skin (pruritus)”, “fainting or dizziness” and feeling “washed out or drained” were the most prevalent symptoms recorded in the SPKD subscale. These symptoms have been previously reported in systematic reviews [51, 52]. Additionally, in support of this observation, a US study reported the negative effects of pruritus on the HRQOL of patients on maintenance hemodialysis beyond the discomfort caused by the condition [53].

Patients’ ratings of effects of the disease, as measured with the subscale EKD, reached a similar level as a study performed in Colombia [30]. However, it is lower than the scores reported by other studies conducted in different parts of the world [31, 32, 34, 48, 54]. Hemodialysis patients must visit medical facilities three times a week for a total of three to five hours; this interferes with their ability to carry out their daily lives independently including work which may contribute to low HRQOL.

Our findings demonstrated that mean PCS and EKD scores decrease as age increases. This finding is supported by previous studies [27, 28, 31, 55] and may be explained by the reduced physical function, strength, energy and self-care ability of elderly patients that occurs with increased age. Complications including heart disease, anemia, high blood pressure, pulmonary edema, and decreased immune response that leads to infection, are also more likely to occur as age increases, which in turn increases pain and leads to further declines in energy and physical functioning [28]. Conversely, the shorter disease duration among younger patients might explain their higher HRQOL scores.

Female sex was associated with lower ratings of mental health (MCS), in line with previous reports [27, 28, 31]. This could be explained by the various roles women play in society that contribute to physical and mental stress, which have a significantly negative influence on the HRQOL of CKD patients [56]. This finding draws attention to the need to support women, who have more roles and responsibilities at home. Thus, a multidisciplinary team of nephrologists, nurses, social workers, and psychologists must work together to support patients undergoing hemodialysis; particularly women.

Consistent with previous studies [27, 28, 31], our data showed that participants with a higher educational levels scored their physical health (PCS) as better and perceived fewer burdens and effects of their kidney disease. This might be because higher education enhances individuals’ understanding of the value and importance of hemodialysis. In addition, higher educational levels are usually associated with higher income and the ability to afford treatment costs, which improves HRQOL.

Technically, patients’ HRQOL is supposed to improve with more frequent dialysis treatments; however, the opposite was found to be true in this study. This study demonstrated that attending hemodialysis sessions three times a week—which is the standard recommendation—was associated with poorer ratings of physical health (PCS subscale). A similar result was found in a Brazilian study [57], which found that attending hemodialysis sessions was associated with a higher prevalence of symptoms, worsening burdens of kidney disease, and lower PCS scores. Similarly, AlSalmi et al. reported that the HRQOL scores of hemodialysis patients decreased with increasing dialysis frequency [58]. This may be due to the patients’ health condition; severely ill patients may undergo hemodialysis three times a week.

The current study showed a positive relationship between adherence to medication and HRQOL with regard to the EKD subscale, consistent with previous findings [59]. This may indicate that non-adherence to medication prevents ESRD patients from gaining the full benefits of the prescribed medications, which has been associated with increased mortality and hospitalisation, leading to poorer HRQOL [60]. The health care team working in the dialysis unit should emphasise health education and counselling regarding medication adherence to improve patients’ HRQOL.

Social support plays a significant role in improving the HRQOL of hemodialysis patients. In line with other studies, we found that having social support was associated with better HRQOL regarding physical health [61–63]. Dialysis treatment should consider the role of family members’, friends’, and neighbors’ participation by welcoming relatives and inviting family members to the dialysis unit. Additionally, due to the gaps they may fill in patients’ support requirements and the shared experiences of the group members, support groups that include patient’s relatives may prove helpful.

In the present study, normal or high BMI were associated with better HRQOL in all domains. About 20% of the respondents had a BMI > 24 kg/m2, which is the recommended BMI for hemodialysis patients [64]. Similarly, Kalantar-Zadeh et al. reported that a higher BMI was protective against cardiovascular disease among hemodialysis patients, in contrast to the general healthy population [65]. This may decrease the risk of having a cardiovascular comorbidity, which in turn contributes to better HRQOL for the patient. Healthcare professionals working in the dialysis unit should be aware of the recommended BMI for ESRD patients undergoing hemodialysis and provide counselling, as needed, to help them maintain it.

The duration of hemodialysis treatment had a significant impact on patients’ HRQOL. According to this study, a duration of one year and above was associated with low SPKD and EKD scores, which is consistent with a previous study [66]. A similar observation was also made by a group in the US, who found that patients who had been on hemodialysis for a longer duration had lower functional status, more hospital readmissions, and worse HRQOL [56]. This is likely explained by the negative feelings brought by the lifelong nature of the treatment, monotonous living, feeling exhausted and fed up, and an inability to deal with the symptoms of dialysis [54]. Thus, as the duration of hemodialysis increases, patients will continue to experience these adverse effects, which may lower their HRQOL.

### Strengths and limitations

The present study has several strengths. First, it assessed HRQOL using a standardized measure (KDQOL-36) that has been used extensively around the world and validated in Ethiopian patients with ESRD, allowing for comparisons with data from other settings. Second, to the best of our knowledge, this study had the largest sample size (481 participants) in sub-Saharan Africa, including both private and governmental facilities, where the cost of hemodialysis varies greatly. This study has limitations. As it is a cross-sectional study, causal inferences cannot be made between HRQOL and the independent variables. Data were collected through face-to-face interviews, which might have introduced interviewer and social desirability bias into the results. Another limitation could be the use of an already-changed terminology (“ESRD” changed to “kidney failure”) for the purpose of continuity with our previous work.

## Conclusion

The results of the present study show that the HRQOL of hemodialysis patients in Ethiopia is low with regard to physical and mental health as well as kidney-related symptoms. Those reporting worse HRQOL were more likely female, older, lacked education, had poor medication adherence, underwent hemodialysis three times a week, had lower BMI, had undergone hemodialysis treatment for at least one year and had poor social support. The intricacies of hemodialysis therapy, as well as the wide range of factors that affect HRQOL, demand comprehensive multidisciplinary care. Therefore, development of clinical guidelines is recommended to improve patients’ adherence to their prescribed medications. Furthermore, establishing patient support groups and encouraging patients to use the available support resources from family members, neighbors, and friends have the potential to improve patients’ HRQOL. Continued research is recommended for a deeper understanding of how patients with ESRD in Ethiopia experience their treatment and life situation.

## Electronic supplementary material

Below is the link to the electronic supplementary material.

**Additional. file 1**: Table 5. **Additional. file 2**: Table 6

## Data Availability

The datasets generated and/or analysed during the current study are available from the corresponding author on reasonable request.

## List of abbreviations

BKD: Burden of kidney disease
EKD: Effect of kidney disease
ESRD: End-stage renal disease
HRQOL: Health-related quality of life
KDQOL-36: Kidney Disease Quality of Life − 36
MCS: Mental Component Summary
PCS: Physical Component Summary
SF-12: Short Form-12
UAE: United Arab Emirates
SPKD: Symptoms/problem lists of kidney disease
WHO: World Health Organisation
