# Supplementary material for: Health-related quality of life among patients with end-stage renal disease undergoing hemodialysis in Ethiopia: a cross-sectional survey
Source: Health Qual Life Outcomes. 2023 Apr 17;21:36. doi: 10.1186/s12955-023-02117-x (PMC10111728; doi:10.1186/s12955-023-02117-x)
Supplement: Supplementary file 1 — Additional. file 1: Table 5. Additional. file 2: Table 6 [file 12955_2023_2117_MOESM1_ESM.docx]

**Section 5: Questions assessing behavioral factors**

| **Se. no.** | **Cigarettes smoking** |  | | |  | | | |
| --- | --- | --- | --- | --- | --- | --- | --- | --- |
| 501 | Have you ever smoked any cigarettes? | 1.Yes  2.No 🡺 | | | *Skip to 507* | | | |
| 502 | How old were you when you **first started** smoking? | _____Age (years) | | |  | | | |
| 503 | For how long did you smoke cigarettes? | ______years  ______months | | |  | | | |
| 504 | Do you currently smoke cigarettes? | 1.Yes  2.No 🡺 | | | Skip to 507 | | | |
| 505 | For how many days per week do you usually smoke cigarette? | _____days | | |  | | | |
| 506 | On average, how many sticks of cigarette do you smoke a day? | _____number | | | |  | | |
| 507 | Is there any family member who smokes cigarette in your home? | 1.Yes  2.No | | |  | | | |
| **Khat use** | | | | | | | | |
| 508 | Have you ever chewed Khat? | 1.Yes  2.No | | | Skip to 512 | | | |
| 509 | Do you currently chew Khat? | 1.Yes  2.No | | | Skip to 512 | | | |
| 510 | During the past 12 months, how frequently did you chew Khat? | 1. Daily 2. 5-6 days per week 3. 3-4 days per week 4. 1-2 days per week 5. 1-3 days per month 6. Less than once a month | | | | | | |
| 511 | When you chewed, on average, **how many** grams of Khat did you use? | ____grams | | | | | | |
| **Alcohol consumption** | |  | | |  | | | |
| 512 | Have you ever consumed any alcohol-containing drinks such as tella, tej, local areke, beer or others? | 1.Yes  2.No 🡪 | | | Skip to Q 601 | | | |
| 513 | For how long did you drink alcohol? | ______year/s  --------months | | |  | | | |
| 514 | Have you consumed any alcoholic drinks within the **past** 12 months? | 1. Yes 2. No | | | Skip to Q 601 | | | |
| 515 | During the **past** 12 months, on how many **days** did you have at least one alcoholic drink? | 1.Daily  2. 5-6 days per week  3. 3-4 days per week  4. 1-2 days per week  5. 1-3 days per month  6. Less than one a month | | | | | |  |
| 516 | During the **past** 12 months when you drunk an alcohol, **on average**, how many **drinks** did you have during one drinking occasion? | **Type** | | **Amount** | | | | |
|  |  | Tella | _____Tasa(ml)  _____Bircheko(ml)  _____Birlie(ml)  _____Wancha(ml) | | | | | |
|  |  | Tej | ______Birlie | | | | | |
|  |  | Local Areki | ______melekia | | | | | |
|  |  | Beer | _______Bottle | | | | | |
|  |  | Other | ________ | | | | | |
| 517 | During the past 12 months, when you consumed an alcoholic drink, how often was it with meals? | 1.Usually  2.Sometimes  3.Rarely  4.Never | | | | |  | |
